# A Deep Learning Approach to Refine the Identification of High-Quality Clinical Research Articles From the Biomedical Literature: Protocol for Algorithm Development and Validation

**DOI:** 10.2196/29398

**Published:** 2021-11-29

**Authors:** Wael Abdelkader, Tamara Navarro, Rick Parrish, Chris Cotoi, Federico Germini, Lori-Ann Linkins, Alfonso Iorio, R Brian Haynes, Sophia Ananiadou, Lingyang Chu, Cynthia Lokker

**Affiliations:** 1 Health Information Research Unit Department of Health Research Methods, Evidence, and Impact McMaster University Hamilton, ON Canada; 2 Department of Medicine McMaster University Hamilton, ON Canada; 3 Department of Computer Science University of Manchester Manchester United Kingdom; 4 The Alan Turing Institute London United Kingdom; 5 Department of Computing and Software Faculty of Engineering McMaster University Hamilton, ON Canada

**Keywords:** bioinformatics, machine learning, evidence-based medicine, literature retrieval, medical informatics, natural language processing, NLP, biomedical, literature, literature surveillance, model development

## Abstract

**Background:**

A barrier to practicing evidence-based medicine is the rapidly increasing body of biomedical literature. Use of method terms to limit the search can help reduce the burden of screening articles for clinical relevance; however, such terms are limited by their partial dependence on indexing terms and usually produce low precision, especially when high sensitivity is required. Machine learning has been applied to the identification of high-quality literature with the potential to achieve high precision without sacrificing sensitivity. The use of artificial intelligence has shown promise to improve the efficiency of identifying sound evidence.

**Objective:**

The primary objective of this research is to derive and validate deep learning machine models using iterations of Bidirectional Encoder Representations from Transformers (BERT) to retrieve high-quality, high-relevance evidence for clinical consideration from the biomedical literature.

**Methods:**

Using the HuggingFace Transformers library, we will experiment with variations of BERT models, including BERT, BioBERT, BlueBERT, and PubMedBERT, to determine which have the best performance in article identification based on quality criteria. Our experiments will utilize a large data set of over 150,000 PubMed citations from 2012 to 2020 that have been manually labeled based on their methodological rigor for clinical use. We will evaluate and report on the performance of the classifiers in categorizing articles based on their likelihood of meeting quality criteria. We will report fine-tuning hyperparameters for each model, as well as their performance metrics, including recall (sensitivity), specificity, precision, accuracy, F-score, the number of articles that need to be read before finding one that is positive (meets criteria), and classification probability scores.

**Results:**

Initial model development is underway, with further development planned for early 2022. Performance testing is expected to star in February 2022. Results will be published in 2022.

**Conclusions:**

The experiments will aim to improve the precision of retrieving high-quality articles by applying a machine learning classifier to PubMed searching.

**International Registered Report Identifier (IRRID):**

DERR1-10.2196/29398

## Introduction

### Background

The biomedical literature grows exponentially every year. According to the latest National Library of Medicine statistical report, more than 1.5 million new citations were indexed in PubMed in 2020 alone [1]. This high volume of literature is fed by the publication of at least 1 new article every 26 seconds [2] and 95 clinical trials per day [3]. Nevertheless, only 1% of published clinical studies meet the criteria for high scientific quality for use in health care decisions [4], driving the need for efficient and accurate approaches to identify clinical studies that have been conducted with methodological rigor.

Methodological search filters like PubMed Clinical Queries [5] are considered the cornerstone for information retrieval in evidence-based practice [6]. These and other search filters have been developed using a diagnostic testing procedure [7] to optimize sensitivity or specificity, or the best balance between the two, for such clinical study categories as treatment, diagnosis, prognosis, etiology, and clinical prediction guides [8]. Some search filters are limited by their partial reliance on MeSH (Medical Subject Headings) indexing terms, as it can take up to a year for articles to be indexed in MEDLINE [9]. Despite having highly sensitive search filters, with an aim to optimize specificity—essentially returning the most likely relevant articles while reducing the need to assess off-target articles—they also return large numbers of articles that are not on target.

Most search filters have been developed using databases of articles that have been tagged for clinical category and methodological rigor and using a diagnostic test approach to detect true and false, positive (on-target) and negative (off-target) articles [10]. The development of such a gold standard is costly and time-consuming, requiring highly trained staff. The Clinical Hedges database, developed at McMaster University, has been used as the gold standard for new search strategy development [11-14].

Machine learning is a subset of artificial intelligence referring to the application of computational methods to improve performance or achieve precise predictions via experience. Experience, in this context, is the information given to the machine for analysis [15]. Machine learning applications in the biomedical literature have been explored by many researchers over the years. In 2007, Yoo and colleagues [16] applied a novel machine learning approach for document clustering and text summarization, producing a textual summary of the information by automatically extracting the most relevant text content from a document cluster. Machine learning has also been applied to the ranking of biomedical publications. In 2009, the MedlineRanker webserver ranked citations in a data set based on their relevance to a given topic [17]. Other applications of machine learning include accurately predicting the citation count of a given article at the time of its publication to determine its scientific impact using a support vector machine (SVM) context–based classifier [18] and automating the systematic review screening process to decrease the screening workload [19]. For example, Miwa and colleagues [20] used an SVM pool–based active machine learning model to classify articles as relevant for inclusion in a systematic review. Miwa et al's [20] experiment used “certainty” as a criterion for article selection, which is effective in dealing with imbalanced data sets. An improvement in topic detection was proposed by Hashimoto et al [21] as they used a neural network model based on paragraph vectors capturing semantic similarities between words and documents. Paragraph vectors can accurately determine semantic relatedness between textual units of varying lengths, that is, words, phrases, and longer sequences (eg, sentences, paragraphs, and documents). Methods that consider factors such as word order within the text yield superior performance [21].

Recent advancement in natural language processing (NLP) is attributed to the development of pretrained language models (PTLMs) [22]. PTLMs transfer learning from training on one data set to the performance of different NLP functions on a new data set [22,23]. PTLMs provide more stable predictions and better model generalization [24]. PTLMs are applied using one of two main strategies: feature-based or fine-tuning models [23]. Feature-based approaches use task-specific architectures that include the pretrained representations as additional features, such as Embedding from Language Models (ELMo) [22,25]. Fine-tuning approaches attempt to pretrain the language model using general-domain text, then fine-tune the model on the target data and target task [22]. Fine-tuning language models are considered the mainstream for PTLM adaption [22]. Examples of fine-tuning approaches are Universal Language Model Fine-Tuning [26] and Bidirectional Encoder Representations from Transformers (BERT) [23]. The bidirectional approach used in BERT improves its performance in understanding text context over other PTLMs, making it the state-of-the-art model. BERT can be used for multiple NLP tasks, including text summarization, retrieval, question answers, named entity recognition, and document classification [27].

Over the past two decades, machine learning has been applied to classify the biomedical literature based on methodological rigor and evidence quality. For such classification tasks, supervised machine learning approaches in which the training data is labeled based on a selected high-quality standard are most commonly used [3,28-32]. The first reported experiments to classify biomedical literature based on quality by Aphinyanaphongs and colleagues relied on the American College of Physicians Journal Club as their high-quality training standard and used a supervised SVM as a classifier [28-30]. The most recent study to classify high-quality articles was conducted by Afzal and colleagues [31] and applied an artificial neural network using data gathered from the Cochrane Library as their high-quality standard. By using supervised approaches, the model development was informed by decisions made by the researchers.

### Objectives

The primary objective of this research is to derive and validate deep learning models using variations of BERT to retrieve high-quality, high-relevance evidence for clinical consideration from the biomedical literature; models will be trained using a large, tagged database of high-quality, high-relevance clinical articles.

## Methods

### Quality Standard Derivation

At McMaster University, the Health Information Research Unit (HiRU) has an established reputation for retrieval, appraisal, classification, organization, and dissemination of health-related research. Through the Knowledge Refinery, the unit daily screens research studies from over 120 clinical journals and identifies those that meet methodological rigor for original studies, systematic reviews, pooled original studies, and evidence-based guidelines within the categories of treatment, primary prevention, diagnosis, harm from medical interventions, economics, prognosis, clinical prediction, and quality improvement [33]. The steps in the process include the initial filtering of all journal articles using highly sensitive search filters (>99%) developed by HiRU to identify articles that fit the categories named above. This filtered subset is then manually reviewed by skilled research associates and a clinical editor. In this project, “rigor” is defined as meeting all the methodological criteria explicitly described on the HiRU website and in Multimedia Appendix 1 [34]. The process of selecting clinically relevant articles is further described by Haynes et al [35], and the high reliability of the critical appraisal step has been documented with a kappa value of over 80% for all categories of articles [36].

The Premium Literature Service (PLUS) process is based on scientific principles for critical appraisal of the medical literature to support evidence-based medicine, combined with multiple ratings of clinical relevance by a worldwide network of practicing health care professionals. Segments of the database have been used many times to test various machine learning approaches, including deep learning [3]. A vast community of >4000 clinicians then rate methodologically rigorous articles for clinical relevance and newsworthiness [37]. The resulting PLUS database contains a distillation of the most reliable and relevant published clinical research [38].

### Data Set

The data used is the Critical Appraisal Process (CAP) data set, which consists of the titles and abstracts of 155,679 articles published between 2012 to 2020, identified by means of their PubMed identifier and manually labeled by research associates as those that “fulfilled” methodological rigor criteria (n=30,035) or “failed” to meet methodological rigor criteria (n=125,644). The data set will be randomly split into 80% for training, 10% for validation, and 10% for testing. Along with being unbalanced, the CAP data set is large and computationally challenging for deep learning model development. To overcome this limitation, we will first convert the data set into multiple balanced subsets, then independently train one model per each of the balanced subsets and use ensembling techniques [39-42] to combine the independently trained models into a better model with more robust performance.

### Machine Learning Experiment

Using Python (Python Software Foundation), we will build our models using the HuggingFace Transformers library [43]. HuggingFace is an open-source NLP and artificial intelligence model hub that provides accessible and implementable state-of-the-art models to the community [44]. Using PTLMs available within the HuggingFace Transformers library, we will experiment with variations of BERT models to determine which have the best performance in article classification. These will include BERT [23], BioBERT [45], BlueBERT [46], and PubMedBERT [47]. These models differ in the pretraining text domain. Pretraining a biomedical BERT model follows a mixed-domain pretraining that initializes with standard BERT pretraining using text data from BookCorpus [48] and English Wikipedia (Wikimedia Foundation) [23], followed by continuous pretraining using biomedical text. BioBERT is pretrained using PubMed abstracts and PubMed Central full-text articles [45], while BlueBERT is pretrained using PubMed text and clinical notes from MIMIC-III (Medical Information Mart for Intensive Care) [46]. PubMedBERT is pretrained using domain-specific text data from a collection of 14 million PubMed abstracts, which were downloaded in February 2020, with abstracts under 128 words removed [47]. Our selection of these models was guided by their availability within the HuggingFace repository and their reported performance in the Biomedical Language Understanding and Reasoning Benchmark leaderboard [47,49].

For the top-performing model that maintains sensitivity >98%, we plan to prospectively validate its real-world performance in the McMaster PLUS reading process. A random sample of incoming articles that are classified as failed articles will be allocated to research staff blinded to the model determination.

To evaluate the performance of machine learning models, we will report the sensitivity (recall), specificity, accuracy, precision, the number of articles that need to be read before finding one that is positive, and F-score (harmonic mean of recall and precision metrics [50]) (Table 1). We will report the probability score threshold, with corresponding 95% CIs, for each model. The machine learning models return a probability score for each article that represents the probability that the article is of high quality, and ranges from 0 (does not meet criteria) to 1 (meets criteria). For a given article, the probability will vary depending on the composition of the model. To prospectively validate the performance of the best model, we will report the same diagnostic characteristics for prospective validation of the model. Fine-tuning hyperparameter settings (number of epochs, learning rate, batch size, and number of random seeds) of the selected models for validation will be reported.

**Table 1:** Definitions and formulas pertaining to performance metrics.

Measure	Definition	Formula
Recall (sensitivity)	The proportion of correctly identified positive articles fulfilling criteria among those predicted to be positive	TP^a^/(TP+FN^b^)
Specificity	The proportion of articles correctly identified as not meeting criteria among those predicted as negative	TN^c^/(TN+FP^d^)
Precision	The proportion of correctly identified positives among all classified positives	TP/(TP+FP)
F-measure	Harmonic mean of precision and recall	2 × ([precision × recall]/[precision + recall])
Accuracy	The number of correctly predicted documents out of all classified documents	(TP+TN)/(TP+FP+FN+TN)
Number needed to read	The number of articles that need to be read before finding one that is positive (meets criteria)	1/precision

^a^TP: true positive.

^b^FN: false negative.

^c^TN: true negative.

^d^FP: false positive.

## Results

Initial model development is underway, with further development planned for early 2022. Performance testing is expected to start in February 2022. Results will be published in 2022.

## Discussion

BERT is considered the state-of-the-art model for NLP. To our knowledge, this is the first experiment to investigate the use of PTLMs in the identification of high-quality articles from the biomedical literature [51]. Our study leverages a large data set of over 150,000 citations that have been manually tagged by experienced research associates, making it one of the few reliable sources for training machine learning models to identify high-quality clinical literature [50]. Our application and analysis of BERT models may provide a better performing automation model suitable for incorporation in literature surveillance processes at HiRU and elsewhere.

Artificial intelligence and machine learning applications are complex and known for their black-box nature, providing predictions without enough explanation [52]. Besides the accurate prediction and the decrease in workload, trust in algorithmic decisions is essential, especially in medicine and health care research [53]. To overcome the lack of transparency, interpreting machine learning models and their decision-making process has become a growing focus among academic and industrial machine learning experts [54]. Next steps include interpreting the decisions made by the model. This would allow us to understand the justification behind model decision-making [55].

## Appendix

Multimedia Appendix 1 Inclusion criteria for articles meeting methodological rigor.

## Abbreviations

BERT: Bidirectional Encoder Representations from Transformers
CAP: Critical Appraisal Process
HiRU: Health Information Research Unit
MeSH: Medical Subject Headings
MIMIC-III: Medical Information Mart for Intensive Care
NLP: natural language processing
PLUS: Premium Literature Service
PTLM: pretrained language model
SVM: support vector machine
