# Supplementary material for: A Deep Learning Approach to Refine the Identification of High-Quality Clinical Research Articles From the Biomedical Literature: Protocol for Algorithm Development and Validation
Source: JMIR Res Protoc. 2021 Nov 29;10(11):e29398. doi: 10.2196/29398 (PMC8669577; doi:10.2196/29398)
Supplement: Multimedia Appendix 1 [file resprot_v10i11e29398_app1.docx]

**Appendix 1**

**Inclusion criteria for articles meeting methodological rigor**

**from https://hiru.mcmaster.ca/hiru/InclusionCriteria.html**

**Basic** criteria for original studies, systematic reviews, pooled original studies, and evidence-based guidelines:

- in English
- about humans
- about topics that are important to the clinical practice of medicine, nursing, rehabilitation, and other health professions, other than descriptive studies of prevalence
- analysis of each article consistent with the study question.

Studies of **prevention or treatment** must meet these additional criteria:

- random allocation of participants to comparison groups
- ≥ 10 patients per group (assessed for outcome)
- primary outcome(s) assessed in ≥ 80% of those randomized
- outcome measure of known or probable clinical importance
- subgroup analyses must be preplanned, with groups analyzed as they were randomized; analyses must test for interaction between ≥ 2 subgroups.

Studies of **diagnosis** must meet these additional criteria:

- inclusion of a spectrum of participants, all suspected of having the diease, with some, but not all, found to have the disease of interest after diagnostic testing
- inclusion of ≥ 100 participants, with ≥ 50 participants with the disease and ≥ 50 participants without the disease
- objective diagnostic ("gold") standard (e.g., laboratory test not requiring interpretation) OR current clinical standard for diagnosis (e.g., a venogram for deep venous thrombosis), preferably with documentation of reproducible criteria for subjectively interpreted diagnostic standard (i.e., report of statistically significant measure of agreement beyond chance among observers)
- each participant must receive both the new test and some form of the diagnostic standard
- interpretation of diagnostic standard without knowledge of test result
- interpretation of test without knowledge of diagnostic standard result
- diagnostic test characteristics reported.

Diagnostic tests may also be tested in randomized trials, in which case the criteria for prevention or treatment apply.

Studies of **prognosis** must meet these additional criteria:

- inception cohort of patients at a similar and early point in the course of a disease or condition, all initially free of the outcome of interest
- prospective standardized data collection
- ≥ 80% follow-up until the occurrence of a major study endpoint or to the end of the study.

Studies of **clinical prediction guides** must meet these additional criteria:

- purpose is to validate or compare a rule/index/scale/model that combines ≥ 2 factors into some type of score/ranking that assigns individual patients to different levels of risk for a specific outcome (diagnosis, prognosis, treatment responsiveness) based on the presence/absence of these factors
- data for the prediction guide must be available before data on the outcome that it is predicting
- the guide must be generated in one or more sets of real (not hypothetical) patients (derivation or development cohort)
- the guide must be validated in another set of real (not hypothetical) patients (validation cohort); internal bootstrapping is not acceptable as validation
  - studies validating a previously derived clinical prediction guide should explicitly state that the derivation was done in a separate patient cohort
  - prediction guides developed using individual patient data from > 1 study do not require separate validation
- study must provide information on how to apply the prediction guide in individual patients or cite a reference to this information.

Studies of **etiology of harm from medical interventions** must meet these additional criteria:

- explicit purpose is to assess adverse effects of an intervention
- prospective standardized data collection with clearly identified comparison groups for those at risk for the outcome of interest
- groups are matched or analyses adjusted to create comparable groups (e.g., quasi-randomized controlled trial, nonrandomized controlled trial, cohort study with case-by-case matching or statistical adjustment to create comparable groups, nested case–control study)
- blinding (masking) of observers of outcomes to exposures (criterion assumed to be met if outcome is objective, e.g., all-cause mortality or objective test)
- if harm reported, relative risk (RR) or hazard ratio (HR) or equivalent ≥ 2.0, with a lower 95% CI that excludes 1.5
- if no harm reported, upper 95% CI of RR or HR or equivalent excludes 1.5.

Randomized controlled trials assessing adverse effects are evaluated using criteria for studies of prevention or treatment.

Studies of **quality improvement or continuing education** must meet these additional criteria:

- random allocation of participants or units to comparison groups
- ≥ 10 patients per group (assessed for outcome)
- ≥ 1 specified outcome assessed in ≥ 80% of those randomized at ≥ 1 follow-up point
- outcome measure of known or probable clinical or educational importance
- subgroup analyses must be preplanned, with groups analyzed as they were randomized analysis must test for interaction between ≥ 2 subgroups.

Studies of the **economics** of health care programs or interventions must meet these additional criteria:

- alternate diagnostic or therapeutic services or quality improvement activities must be compared on the basis of both the outcomes produced (effectiveness) and resources consumed (costs) in real patients
- evidence of both effectiveness and costs reported in a single randomized controlled trial that passes criteria for prevention or treatment
- results must be presented in terms of the incremental or additional costs and outcomes of one intervention over another.

**Systematic review** articles must meet these additional criteria:

- explicit statement of the clinical topic
- identifiable description of the methods, including the databases searched and inclusion and exclusion criteria for selecting articles for detailed review; reviews of treatment, primary prevention, quality improvement, or economics must search for RCTs; reviews of prognosis must have "inception cohort" as an inclusion criteria
- > 1 major database searched
- number of articles retrieved/reviewed and the number passed/included must be reported.

**Pooled original studies** must meet these additional criteria:

- analysis in which patient-level data are pooled from ≥ 2 studies/cohorts/sources to assess a question related to one of the study categories but article DOES NOT meet criteria for a systematic review

**Evidence-based guidelines** must meet these additional criteria:

- the Guideline must be based on a published systematic review that passes our current crtieria for a Review
- methods and findings of the systematic review may be reported within the Guideline document or in a separate document that accompanies the Guideline or is cited in the Guideline and is accessible
- evidence underpinning the recommendations must be reported (e.g., citations of studies, estimates of effect, etc.)
- the strength of the evidence (such as GRADE) for the recommendations must be reported
